# Intelligence Over Time in Children with Neurofibromatosis Type 1 Based on a Structured Natural History-Study

**DOI:** 10.1177/08830738261416621

**Published:** 2026-02-17

**Authors:** Sandra van Abeelen, Jos G. M. Hendriksen, Anton de Louw, Marie Claire Y. de Wit, Pieter F. A. de Nijs, Rianne Oostenbrink, André B. Rietman

**Affiliations:** 1 3804 Kempenhaeghe, Centre for Neurological Learning Disabilities, Heeze, the Netherlands; 2 School for Mental Health and Neuroscience, Maastricht University, Maastricht, the Netherlands; 3 ENCORE Expertise Centre for Neurodevelopmental Disorders, 97759 Erasmus MC Sophia Children's Hospital, Rotterdam, the Netherlands; 4 Department of Pediatric Neurology, 97759 Erasmus MC Sophia Children's Hospital, Rotterdam, the Netherlands; 5 Department of Child and Adolescent Psychiatry/Psychology, 97759 Erasmus MC Sophia Children's Hospital, Rotterdam, the Netherlands; 6 Department of General Pediatrics, 97759 Erasmus MC Sophia Children's Hospital, Rotterdam, the Netherlands

**Keywords:** ADHD, children, intelligence, longitudinal, neurofibromatosis type 1

## Abstract

This study examines the course of intelligence in children with neurofibromatosis type 1 (NF1) and factors influencing changes. In this cross-sectional and longitudinal study, 397 children were assessed at ages 3, 6, 11, and 15 years using a neuropsychological test battery. Comparisons of demographics and scores were conducted across and within cross-sectional and longitudinal groups. Cross-sectionally, 15-year-olds outperformed 11-year-olds on Performance IQ (PIQ). A reduced PIQ at age 11 years was found that appeared to recover by age 15 years in the longitudinal group. The mother's level of education, the mode of inheritance, or attention-deficit hyperactivity disorder were not predictive of these changes. Overall, intelligence remains stable in children with NF1 between 3 and 15 years of age. The gap between PIQ and VIQ decreases over time. Neurocognitive monitoring is recommended during the critical period between ages 6 and 11 years due to risks of attention and learning issues.

Neurofibromatosis type 1 (NF1) is a genetic disorder, with an estimated prevalence of 1 in 2052.^1,2^ About half of the NF1 cases result from a de novo inheritance on the NF1-gene that encodes for neurofibromin on chromosome 17q11.2.^3^ Clinical diagnostic criteria include multiple café-au-lait spots, axillary or groin freckling, neurofibromas, optic pathway glioma, bone dysplasia, and Lisch nodules.^4,5^ Additionally, significant difficulties in language, reading, visuospatial skills, motor function, executive function, attention, behavior, emotion, and social skills are reported, particularly in children and adolescents.^6–8^

## Intellectual Development in NF1 in Previous Longitudinal Studies

In general, the mean IQ scores of children with NF1 show a left shift of 10 points compared with the general population. IQ scores therefore generally fall within the average range for Full Scale IQ, Verbal IQ (VIQ), and Performance IQ (PIQ).^9^ The number of people with NF1 with an intellectual disability (Wechsler Full-scale Intelligence Quotient [FSIQ] below 70) is estimated at 4% and double the size within the general population.^10^

Several longitudinal studies have described the course of intellectual development in children with NF1 between the primary school years and adolescence. Young children of age 5-14 months show an increase in developmental abilities over time, similar to controls.^11^ A prospective longitudinal study in which the developmental trajectory of toddlers (n = 39) was compared to a control group showed that later general intelligence can be predicted by early mental function.^12^ Children with NF1 follow the same developmental trajectory as healthy peers. They show lower scores on a developmental scale over time at 21, 30, and 40 months of age. Longitudinal follow-up of the parent-reported developmental course of children with NF1 (n = 43) between 0 and 2 until 8 years of age, showed that developmental milestones appear to be reached increasingly later over time.^13^

Results from longitudinal analysis in 19 children with NF1 aged 6-16 years reported similar growth on different age-appropriate Wechsler intelligence tests compared with their siblings.^14^ A longitudinal history study in children aged 6-18 years with NF1 and at least 1 plexiform neurofibroma, found no age effect on VIQ, whereas PIQ significantly increased in older children (>14 years), decreased in younger children (<10 years), and remained relatively stable for children aged 10-14 years.^15^ Age-related changes in IQ were found to be comparable between NF1 and a control group in a prospective longitudinal follow-up study of 32 NF1 children (aged 8-16 years) and 11 unaffected sibling controls using a Wechsler test.^16^ FSIQ gains were 8.8 points in adolescents and 4.2 points in children aged 8-10. This study was extended to include an age group of patients aged 25-35 years.^17^ Over an 18-year study period, a significant increase in cognitive function in patients with NF1 was found (n = 18).

All of these studies do not exclusively use the Wechsler intelligence scales and primarily involve small groups of convenience samples of children in a limited age group, rather than covering the entire age group of children.

## Verbal and Performance Intelligence

There are mixed findings regarding observed patterns of strengths and weaknesses on measures of intelligence. Wechsler FSIQ can be divided in Verbal IQ (VIQ) and Performance IQ (PIQ). In a longitudinal study with 114 German children with NF1 aged 6-17 years using the German Wechsler Intelligence Scale for Children, third version, higher scores on the VIQ were found, compared to the PIQ.^18^ These findings were confirmed in a retrospective cross-sectional study in which 27 participants aged 6-16 years were examined using the WISC fourth version. Children with NF1 scored significantly lower on PIQ, processing speed, and working memory, compared to VIQ.^19,20^ In contrast, other studies^9,21^ did not find differences between VIQ and PIQ in children with NF1.

## Possible Contributors to Observed Cognitive Functioning

A diagnosis of attention-deficit hyperactivity disorder (ADHD) is common in about one-third of the children with NF1 and may affect cognition and executive function.^7,22^ ADHD consists of 3 subtypes: the inattentive, the hyperactive/impulsive, and the combined type according to the criteria of the *Diagnostic and Statistical Manual of Mental Disorders, Fourth Edition, Text Revision* (*DSM-IV-TR*).^23^ Children with NF1 are more likely to be diagnosed with either inattentive-type or combined-type ADHD.^24^ Not much longitudinal research has been done on the influence of ADHD on intellectual development in children with NF1. In a study with 111 children between the ages of 6 and 12 years, attention problems and ADHD were potential risk factors for lower IQ in children with NF1.^25^

In addition to ADHD, other variables may play a role in the course of intellectual development in children with NF1. There is conflicting evidence in the literature regarding the influence of sex on intellectual functioning in children with NF1. One study in a small group of children found an effect of sex on intellectual ability, with a lower IQ in boys with NF1.^21^ In a longitudinal study with 88 children with NF1, no sex differences were found.^15^ This study also found that children of parents with higher levels of education exhibited higher scores on Verbal and Performance IQ. The relevance of this proxy of socioeconomic status lies in its strong relationship with cognitive ability from early childhood.^26,27^ A longitudinal study showed a discrepancy between familial NF1 and spontaneous NF1 in 88 children with plexiform neurofibromas between 6 and 18 years of age.^15^ Children with familial NF1 showed a declining trend in performance IQ over 6 years. The group with a de novo variant showed relatively stable cognitive functioning.

## Aim of the Current Study

To date, there is a shortage of prospective studies into the course of intellectual development in children with NF1. Results concerning changes in intelligence over time are conflicting, sample sizes are mostly small and most results have been based on cross-sectional or retrospective designs.

Longitudinal research is needed to give us more insight into the course of intellectual development in children with NF1 and the variables associated with this development. It is also not sufficiently clear whether FSIQ, VIQ, or PIQ develop in the same way in children with NF1.

The current study aimed to describe (1) the course of FSIQ, VIQ, and PIQ in children with NF1 aged 2 years 6 months to 16 years in 3 longitudinal samples, and (2) investigate whether maternal educational level, mode of inheritance, or ADHD are covariables in the association between IQ and age. In this way, we hope to determine which children are at risk for developmental delays or disorders and therefore need to be monitored.

## Materials and Methods

### Study Design and Participants

This study is a prospective cohort study and uses cross-sectional and longitudinal data from charts of children with NF1 in the VOLG study (Dutch acronym for Vroegtijdige Onderkenning Leer- en Gedragsproblemen: “Early recognition of problems in learning and behavior”). Data collection of the VOLG-study was performed with the approval of the Medical Ethics Review Committee Rotterdam on October 31, 2014 (MEC-2015-203), and in accordance with the Declaration of Helsinki.^9^

Children participate in the VOLG study either at the outpatient clinic of the Erasmus Medical Centre (EMC), Sophia Children's Hospital in Rotterdam, or at the outpatient clinic of Kempenhaeghe Centre for Neurological Learning Disabilities (KCNL), both in the Netherlands.

**Figure 1.**
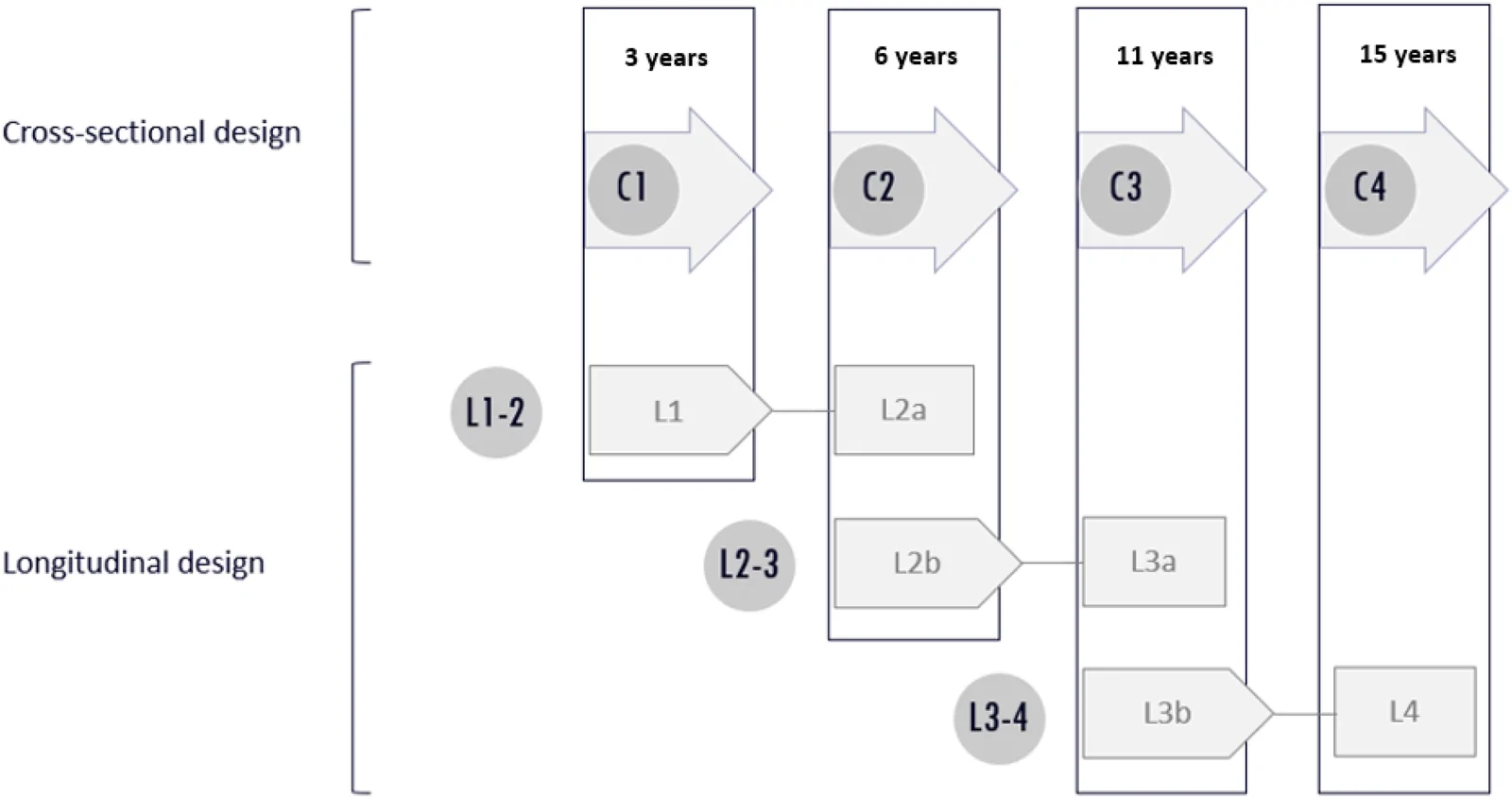
Cross-sectional and longitudinal design. C1 cross-sectional group, 3 years: mean age 3.2 years (SD 0.55); C2 cross-sectional group, 6 years: mean age 5.9 years (SD 0.73); C3 cross-sectional group, 11 years: mean age 11.3 years (SD 0.89); C4 cross-sectional group 15 years: mean age 15.0 years (SD 0.74). L1-2 consists of longitudinal groups L1, 3 years: mean age 3.3 (SD 0.61) and L2a, 6 years: mean age 5.9 (SD 0.7); L2-3 consists of longitudinal groups L2b, 6 years: mean age 6.3 years (SD 0.69) and L3a, 11 years: mean age 10.8 (SD 0.79); L3-4 consists of longitudinal groups L3b, 11 years: mean age 11.3 (SD 0.97) and L4, 15 years: mean age 14.9 years (SD 0.61).

This study consists of a cross-sectional and longitudinal design as is shown in Figure 1. Children with NF1 from ages 2 to 16 years are routinely referred to one or both clinic(s) at varying ages. They were referred for standard care, mostly by a pediatrician. According to the Dutch standard of care for patients with NF1 (Neurofibromatose Vereniging Nederland “Dutch Neurofibromatosis Association,” 2015), several time points for neuropsychological assessment are scheduled at key ages in development and education: T1 between ages 2 years 6 months and 4 years, T2 between ages 5 and 7 years, T3 between ages 10 and 13 years, and T4 between ages 14 and 16 years. The above neuropsychological assessment times are referred to as T1: 3 years, T2: 6 years, T3: 11 years, and T4: 15 years of age (see Figure 1).

The children who participated in this study were evaluated from 2010 to 2020. A standardized test battery was administered by experienced neuropsychologists. This consists of an age-appropriate intelligence test and tasks to evaluate neurocognitive domains such as attention, memory, and visual-constructive functioning. Socioeconomic status was derived from the mother's education level^28^ according to the International Standard Classification of Education (ISCED). ISCED categories were combined into (1) Low (ISCED 0-2), (2) Middle (ISCED 3-4), and (3) High (ISCED 5-8) and finally dichotomized into Low-Middle or High.^29^ A clinical diagnosis of ADHD was usually made between the ages of 6 and 11 years, using psychiatric and psychological assessment and DSM-IV or DSM-5 criteria.^23^ Diagnoses were dichotomized as inattentive type and combined or hyperactive/impulsive type. Information on the presence of a chromosomal microdeletion genotype (CMD), familial or de novo inherited, was extracted from patient records.

This study was approved by the Medical Ethical Committee (MEC) of Erasmus MC- Sophia Children's Hospital (MEC-2015-203) and of Kempenhaeghe (MEC-16.30). Informed consent was obtained from parents and from children aged 12 years and older.

### Outcome Measures

Wechsler intelligence scores for FSIQ, VIQ, and PIQ were the main outcome measures (dependent variables) at all time points. From 4 years of age, a score was also calculated on the Processing Speed Index (PSI). Intelligence at T1 was assessed with the Wechsler Preschool and Primary Scale of Intelligence–Third edition, Dutch version (WPPSI-III-NL). At T2, either the WPPSI-III-NL or the Wechsler Intelligence Scale for Children–Third edition, Dutch Version (WISC-III-NL) was used.^30,31^ At T3 and T4, the WISC-III-NL was used. Both intelligence tests have a population mean of 100 and an SD of 15 for IQ of index scores.

The WPPSI-III-NL is a test measuring intelligence in children between the ages of 2 years 6 months and 7 years 11 months. The test consists of 4 core subtests and an optional complementary subtest for the age group 2 years 6 months to 3 years 11 months. In the age groups 4 years to 7 years 11 months, there are 7 core subtests. The instrument determines the cognitive capacity of children using 3 IQ scores for the youngest age group: VIQ, PIQ, and FSIQ. It is possible to add the PSI from the age of 4 years. The internal consistency of the WPPSI-III-NL ranges from acceptable to excellent, with Cronbach α estimated between 0.86 and 0.96 for full-scale IQ. The test-retest reliability is good with a Pearson *r* of 0.84 for full-scale IQ.^30^

The WISC-III-NL measures intelligence in children aged 6 years up to 16 years 11 months. In addition to a VIQ, PIQ, and FSIQ, the WISC-III also contains 3 indexes: Verbal Comprehension Index (VCI), Perceptual Organization Index (POI), and PSI. The internal consistency of the WISC-III-NL is excellent for Verbal IQ (α = 0.92), good for Performance IQ (α = 0.87), and excellent for full-scale IQ (α = 0.94). The test-retest reliability was shown to be good for Verbal IQ (r = 0.87), good for Performance IQ (r = 0.87), and excellent for full-scale IQ (r = 0.91).^31^

The correlations between the WPPSI-III-NL and the WISC-III-NL (ranging from 0.68 to 0.82) provide evidence of content validity and show that these tests measure the same concept, which is general intelligence.

### Inclusion and Exclusion Criteria

Inclusion criteria were a confirmed genetic diagnosis of NF1 or a clinical diagnosis according to Legius.^5^ Patients were included if at least 1 assessment took place at one of the 4 previously described time points (T1-T4).

Exclusion criteria were prematurity (gestational age <34 weeks), symptomatic central nervous system (CNS) pathology (cerebral tumor or optic pathway glioma requiring treatment), deafness or severely impaired vision, insufficient production or comprehension of the Dutch language, and an IQ lower than the range covered by intelligence testing.

### Statistical Analysis

Data were analyzed using SPSS (Statistical Package for the Social Sciences) version 27 (IBM Corp). First, χ^2^ tests were performed to compare the proportions of the cross-sectional and corresponding longitudinal groups on demographic variables (age, sex, mother's level of education [MLE; as a proxy for maternal socioeconomic status], mode of inheritance, and comorbid ADHD. Two-sided independent samples *t* tests were used for comparisons of group means between the cross-sectional and corresponding longitudinal group for continuous, normally distributed variables. A Mann-Whitney *U* test for 2 independent variables was used for non-normally distributed data. The Shapiro-Wilk test was used to control for normal distribution in groups with n <50, the Kolmogorov-Smirnov test was used for the same reason in groups with n >50.

For the cross-sectional part of this study, we compared VIQ, PIQ, and PSI within each cross-sectional cohort. In addition, we compared FSIQ, VIQ, and PIQ at C1 through C4 and PSI at C2 through C4. If a child was seen once, he or she entered the cross-sectional sample.

For the subgroups within sex (male/female), MLE (low/high), mode of inheritance (de novo/familial), and ADHD (yes/no) we looked for differences in IQ scores. Independent samples *t* tests were used for normally distributed data, Wilcoxon signed ranks tests for non-normally distributed data.

In the longitudinal part, the FSIQ, VIQ, PIQ, and PSI scores of the same children at 2 ages were compared in 3 groups: L1-2, L2-3, and L3-4 at 2 different time points. In this part, we used paired samples *t* tests or Wilcoxon signed-rank tests. Children could enter at different times: L1, L2, or L3, depending on the time of referral to one of the outpatient clinics. If a child was seen twice, it was included in the longitudinal group.

Multivariable regression analyses were used to test whether the independent variables of age, MLE, mode of inheritance, or ADHD are predictive for FSIQ, VIQ, PIQ, and PSI in children at L2a, L3a, or L4.

The cutoff level for significance of *P* value was set at α = .05. Effect sizes were calculated using Cohen *d* or *r* with 0.20 interpreted as a small effect size, 0.50 as medium, and 0.80 as large.^32^ Where applicable due to multiple comparisons, a Bonferroni correction has been used.

## Results

### Patient Characteristics

**Figure 2.**
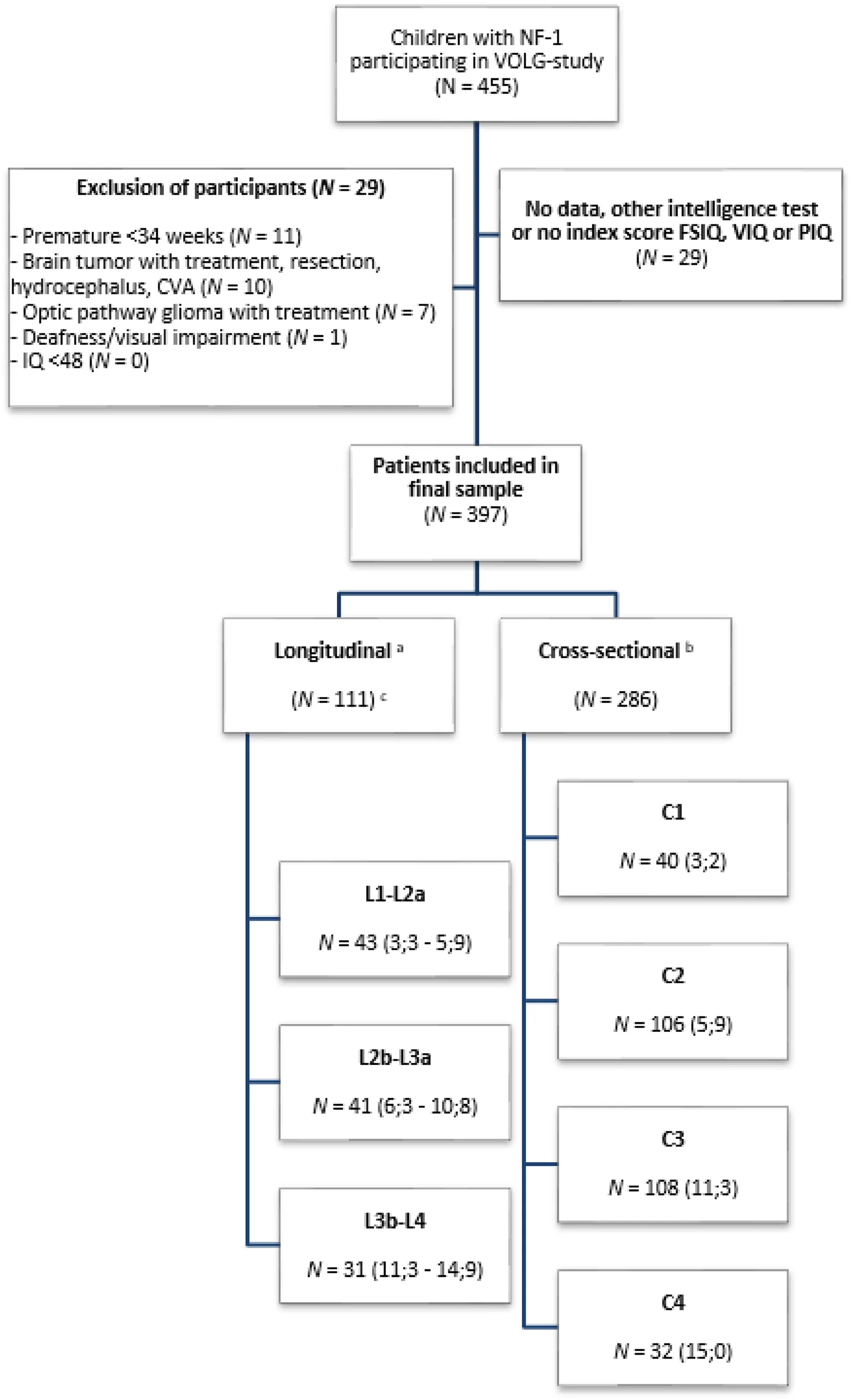
Flow chart of patient inclusion. ^a^Longitudinal groups: L1-L2a consists of longitudinal groups 3 and 6 years; L2b-3a consists of longitudinal groups 6 and 11 years; L3b-L4 consists of longitudinal groups 11 and 15 years. ^b^Cross-sectional groups: C1 cross-sectional group 3 years; C2 cross-sectional group 6 years; C3 cross-sectional group 11 years; C4 cross-sectional group 15 years. ^c^In the longitudinal group, 2 patients were seen at both L1-L2a and L2b-L3a and 2 patients at both L2b-L3a and L3b-L4. Because of this, the total number of patients in the longitudinal group is not 115 (summed) but 111. *N* (Mean ages in years). For longitudinal groups, the mean age is given for both of the age groups that are being compared.

Finally, 455 children were available for the VOLG study. Of these, 29 children were excluded because of prespecified exclusion criteria and 29 children had unavailable data, leaving 397 children eligible for this particular study, of which 386 had a genetic diagnosis. A flowchart of included patients is presented in Figure 2. For a summary of demographic and NF1 characteristics and the presence or absence of a comorbid diagnosis of ADHD in children with NF1, see Table 1. All longitudinal participants have data at 2 points in time, except for 4 children. Two of them were tested at both L1-L2a and L2b-L3a. And another 2 children were tested at both L2b-L3a and L3b-L4 (see Figure 2).

**Table 1.** Characteristics of Children with NF1.

	Longitudinal			Cross-sectional				
	**L1-L2a** ^a^	**L2b-L3a** ^a^	**L3b-L4** ^a^	**Total C**	**C1** ^b^	**C2** ^b^	**C3** ^b^	**C4** ^b^
*N* = 397^c^	n = 43	n = 41	n = 31	n = 286	n = 40	n = 106	n = 108	n = 32
**Demographic characteristics**								
Age (mean, SD)	3.3 (0.61)-5.9 (0.7)	6.3 (0.69)-10.8 (0.79)	11.3 (0.97)-14.9 (0.61)	8.7 (3.78)	3.2 (0.55)	5.9 (0.73)	11.3 (0.89)	15.0 (0.74)
Sex (male n, %)	24 (56)	17 (42)	17 (55)	161 (56)	24 (60)	59 (56)	57 (53)	20 (63)
Mother's level of education (proxy for SES mother, n, %)								
Low/medium	23 (56)	20 (59)	12 (57)	152 (62)	17 (47)	56 (60)	63 (68)	16 (73)
High	18 (44)	14 (41)	9 (43)	93 (38)	19 (53)	37 (40)	30 (32)	6 (27)
Missing	2	7	10	42	4	13	15	10
**NF1 characteristics**								
Mode of inheritance NF1 (n, %)								
Familial	9 (22)	9 (24)	10 (34)	79 (28)	6 (15)	29 (28)	36 (34)	8 (25)
De novo	32 (76)	22 (56)	13 (45)	168 (59)	34 (85)	67 (66)	51 (48)	17 (53)
Unknown	1 (2)	8 (21)	6 (21)	34 (12)	-	6 (6)	19 (18)	7 (22)
Microdeletion	-	-	-	8 (3)	3 (8)	2 (2)	2 (2)	1 (3)
**ADHD**								
No ADHD	31 (72)	17 (42)	21 (68)	179 (63)	34 (85)	62 (59)	62 (57)	21 (66)
ADHD combined/hyperact-imp type	6 (14)	14 (34)	7 (23)	72 (25)	6 (15)	34 (32)	26 (24)	6 (19)
								
ADHD inattentive type	6 (14)	10 (24)	3 (9)	35 (12)	-	10 (9)	20 (19)	5 (15)
Using stimulant medication (n*,* % yes)	–	9 (38)	8 (80)	24 (22)	–	-	20 (44)	4 (36)
Missing	–	–	–	4	-	3	1	-

Abbreviations: ADHD, attention-deficit hyperactivity disorder; NF1, neurofibromatosis type 1; SES, socioeconomic status.

a Longitudinal groups: L1-L2a (3 and 6 years); L2b-L3a (6-11 years); L3b- L4 (11 and 15 years).

b Cross-sectional groups: C1 cross-sectional group 3 years; C2 cross-sectional group 6 years; C3 cross-sectional group 11 years; C4 cross-sectional group 15 years.

c In the longitudinal group, 2 patients were seen at both L1-L2a and L2b-L3a and 2 patients at both L2b-L3a and L3b-L4. Because of this, the total number of patients is 397.

Information regarding the mother's socioeconomic status, represented by her level of education, was incomplete in files of 61 children. No children with microdeletions were present in any of the longitudinal groups.

### Comparison Between Cross-Sectional and Longitudinal Groups

The scores of children from the longitudinal (starting with “L”) and cross-sectional groups (starting with “C”) were analyzed separately for unambiguous interpretation.

The cross-sectional group consisted of 286 children divided into 4 age groups C1, C2, C3, and C4. The longitudinal group consisted of 111 children in total, divided into 4 age groups L1 and L2a (comparison ages 3-6, n = 43); L2b and L3a (comparison ages 6-11, n = 41); L3b and L4 (comparison ages 11-15, n = 31) of approximately the same ages as in C1-C4. L2a and L2b are different children of 6 years old and L3a and L3b are different children of 11 years old (see Table 1). Pairwise comparisons were made between children in the cross-sectional age groups and their longitudinal counterparts.

### Intelligence Within Cross-Sectional Groups

We found a significant difference between VIQ and PIQ in 6- and 11-year-olds (C2 and C3), with small effect sizes (respectively *r* = −0.20 and *d* = 0.45, see Table 2). After correction for multiple comparisons, the difference between the VIQ and PIQ scores was no longer significant for the 6-year-old group (C2).

**Table 2.** Intelligence Within Cross-Sectional Groups.

Cohort (N, Mean Age in Years, SD)	FSIQ	VIQ	PIQ	PSI	*P* ^c^	Effect Size
**C1**^a^ (40, 3 y 2 mo, SD 0.6)	89.23 (16.92)	91.65 (16.52)	89.60 (15.99)	- ^f^	VIQ-PIQ: .407	0.133
**C2** ^a^ (106, 5 y 9 mo, SD 0.7)	89.35 (12.43)	92.78 (14.50)	89.96 (12.47)	90.87 (17.69)	VIQ-PIQ: .040^g^	−0.20 ^e^
Missing^b^	1			4	VIQ-PSI: .326	0.098 ^d^
					PIQ-PSI: .845^g^	−0.02 ^e^
**C3** ^a^ (108, 11 y 3 mo, SD 0.9) Missing ^b^	90.32 (15.80)	93.59 (15.11)	87.55 (16.95)	95.38 (16.38)	VIQ-PIQ: <.001***	0.454 ^d^
				1	VIQ-PSI: .234	−0.116 ^d^
					PIQ-PSI: <.001***	−0.519 ^d^
**C4** ^a^ (32, 15 y, SD 0.7)	90.78 (16.88)	91.59 (17.63)	92.28 (16.53)	95.78 (16.85)	VIQ-PIQ: .802	−0.045 ^d^
					VIQ-PSI: .130	−0.275 ^d^
					PIQ-PSI: .183	0.241 ^d^

Abbreviations: FSIQ, Full-Scale Intelligence Quotient; PIQ, Performance Intelligence Quotient; PSI, Processing Speed Index; VIQ, Verbal Intelligence Quotient

a C1 cross-sectional group 3 years, C2 cross-sectional group 6 years, C3 cross-sectional group 11 years, C4 cross-sectional group 15 years.

b Part of test not administered for unknown reason.

c Significant differences between subscales of intelligence.

d Effect size is represented by Cohen *d*.

e Effect size is represented by *r*.

f PSI could not be administered to children at this age.

g Wilcoxon signed-rank-test was performed because of non-normally distributed variable.

*P < .05. **P < .01. ***P < .001. After correction for multiple comparisons α was reduced to 0.017 (*P* < .05). In groups C2, C3, C4: 0.05/3.

We also found a significant difference between PIQ and PSI in 11-year-olds (C3) with a medium effect size (*d* = −0.519). PIQ scores are roughly 6 to 8 points lower than VIQ and PSI in this group of children. No significant differences were found in children aged 15 years (C4).

When comparing intelligence scores of the subgroups within the background variables (male or female, mode of inheritance, etc), we found 3 significant differences with small effect sizes: in 6-year-olds (C2) males < females on PSI (*P* = .019, *d* = −0.478), in 11-year-olds (C3) familial mode of inheritance < de novo mutation on FSIQ (*P* = .04, *d* = 0.455) and ADHD < no ADHD on PSI (*P* = .034, *d* = 0.420). Multiple comparisons may again have influenced the differences we found. Overall, we were not able to conclude that there were major differences in IQ within the background variables in this sample. We did not perform the same analysis in the longitudinal groups, because of small sample sizes.

### Intelligence Between Cross-Sectional Groups

No significant differences were found when comparing FSIQ, VIQ, PIQ, and PSI between age groups 3-6, 6-11, and 11-15 years (see Supplementary Material, Table S3).

### Intelligence Within Longitudinal Groups

**Figure 3.**
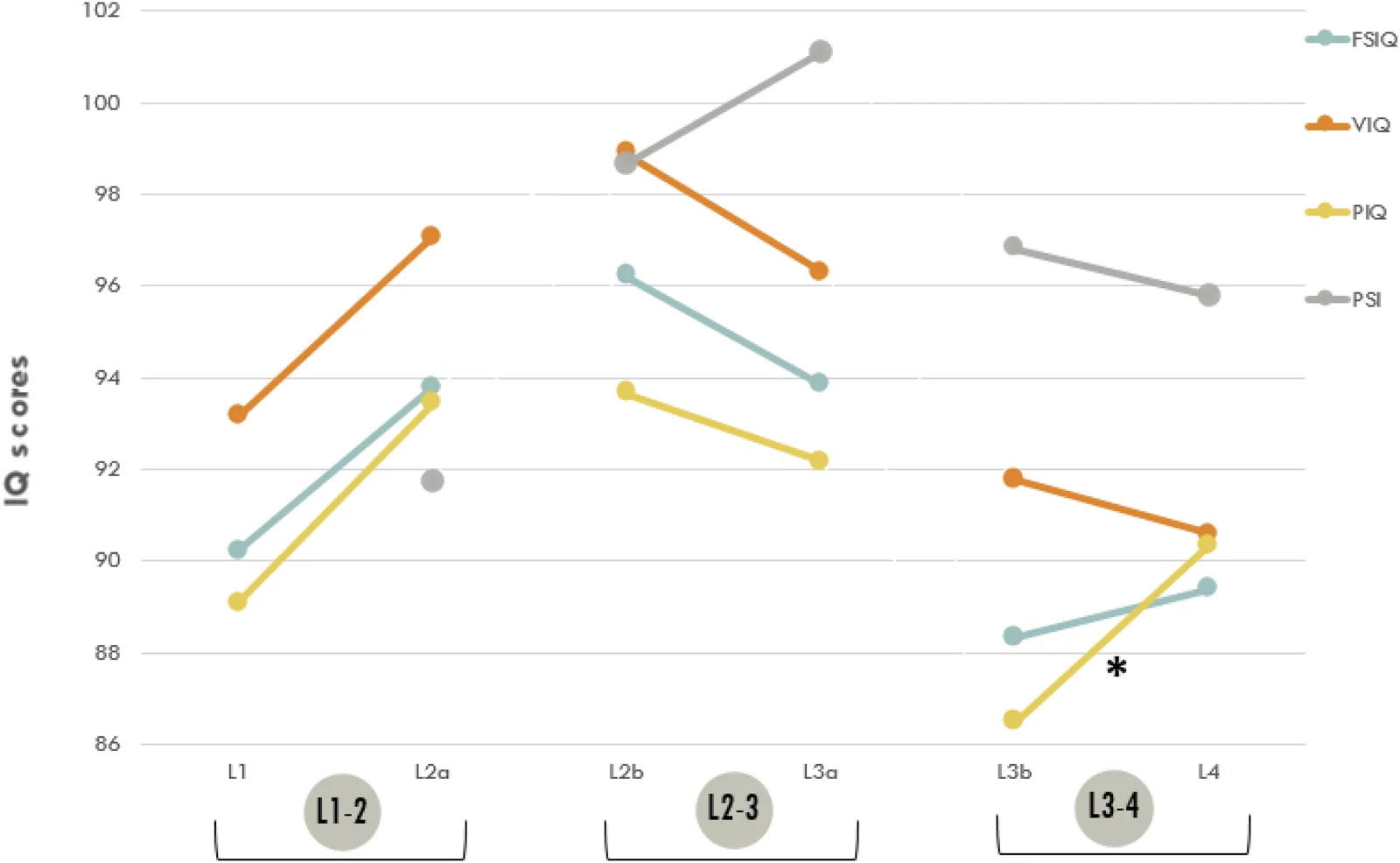
Intelligence in longitudinal groups. Longitudinal groups: L1-2 consists of longitudinal groups L1 and L2a (3 and 6 years); L2-3 consists of longitudinal groups L2b and L3a (6 and 11 years); L3-4 consists of longitudinal groups L3b and L4 (11 and 15 years). *P < .05. **P < .01. ***P < .001. After correction for multiple comparisons α was reduced to 0.017 (*P* < 0.05) for L1-2: 0.05/3 and to 0.013 for L2-3 and L3: 0.05/4.

Figure 3 shows analyses of differences in FSIQ, VIQ, PIQ, and PSI over time. We found an increase in FSIQ (*t* = –2.361, *P* = .023), in VIQ (*t* = –2.370, *P* = .022), and in PIQ (*t* = –2.188, *P* = .034) between 3 years (L1) and 6 years (L2) with small effect sizes. However, after correction for multiple comparisons, these differences were no longer significant. No significant differences were found in FSIQ, VIQ, PIQ, and PSI over time between 6 years (L2) and 11 years (L3). Finally, when exploring the differences between the children aged 11-15 years (L3-4 groups), we observed a significant increase in PIQ between L3b (86.48, SD = 14.2) and L4 (90.35, SD = 17.3, *P* = .017) with a small effect size (Cohen *d* = −0.454), even after correction. For FSIQ, VIQ, and PSI, we found no significant differences.

In addition to comparisons *between* longitudinal groups, comparisons were made *within* longitudinal groups (see Supplementary Material, Table S4). No significant differences were found within children aged 3 years (L1). In 6-year-olds, we found a difference in L2a (n = 43, mean 5 years 9 months) between VIQ (97.07, SD 14.1) and PSI (91.72, SD = 17.0, *P* = .027). The effect size is small (Cohen *d* = 0.350). After correction for multiple comparisons, this significance does not hold. We observed a significant difference for L2b among VIQ (98.88, SD = 13.1) and PIQ (93.66, SD = 15.0, *t* = 2.727, *P* = .009) with a small effect size (Cohen *d* = 0.426). Another significant difference was detected within 11-year-olds (L3a) for PIQ (92.17, SD = 16.4) vs PSI (100.93, SD = 15.2, *P* = <.001; medium effect size: *d* = −0.659). Children at this age (mean = 10 years 8 months) score approximately 9 points higher on PSI compared with their scores on PIQ. Similar to the difference between PIQ and PSI in group L3a, group L3b (n = 31, mean = 11.3) showed a significant difference between PIQ (86.48, SD = 14.2) and PSI (96.81, SD = 12.1, *P* = <.001) with a large effect size (Cohen *d* = −0.935) even after correction. In group L4 (n = 31, mean = 14 years 9 months), a difference was observed between PIQ (90.35, SD = 17.3) and PSI (95.77, SD = 13.7, *P* = .026) with a small effect size (Cohen *d* = −0.421). This difference appeared not significant after correction.

**Figure 4.**
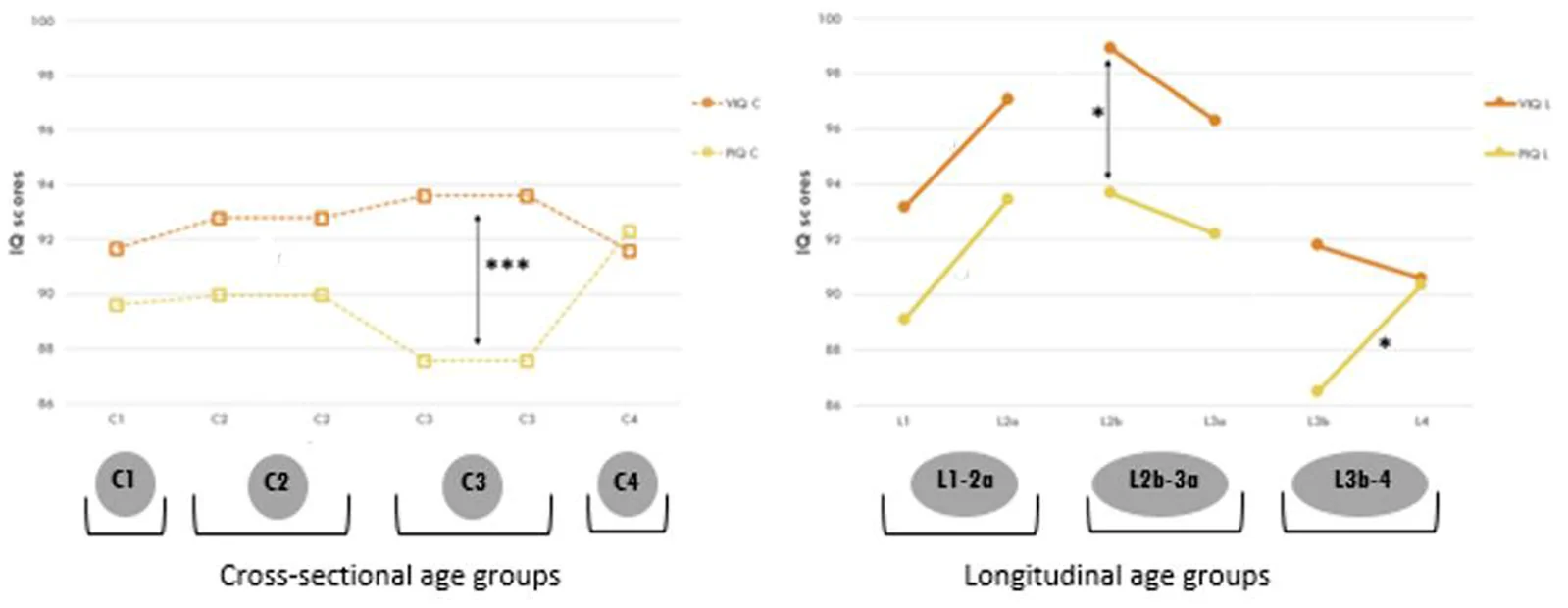
Verbal (VIQ) and Performance IQ (PIQ) in cross-sectional and longitudinal groups. Solid lines represent VIQ and PIQ in the longitudinal groups. Longitudinal groups: L1: 3 years; L2a and L2b: 6 years; L3a and L3b: 11 years; L4: 15 years. The dotted lines represent VIQ and PIQ in the cross-sectional groups. Cross-sectional groups: C1: 3 years; C2: 6 years; C3: 11 years; C4: 15 years. *P < .05. **P < .01. ***P < .001. Represents a significant difference between 2 longitudinal groups (asterisks only) or a significant difference between VIQ and PIQ within a longitudinal or cross-sectional group (vertical arrows). Note: On the *x* axis, groups of children of the same age may be listed twice in the cross-sectional group (eg, C2 and C3), as we are comparing C1 with C2, C2 with C3, and so on. The same applies to the longitudinal group, where we refer to 2 different groups of 6-year-olds (L2a and L2b). Refer to Supplementary Table S4 for data.

Figure 4 shows the course of VIQ and PIQ in the longitudinal groups compared with VIQ and PIQ scores of the cross-sectional groups. Within-group comparisons showed only a significant difference between VIQ (98.88, SD = 13.1) and PIQ (93.66, SD = 15.0, *t* = 2.727, *P* = .009) in longitudinal group L2b. At that age, there was also a significant but smaller difference between VIQ and PIQ in the cross-sectional group C2 (*P* = .040). At age 11 years (C3), we found another significant difference between VIQ and PIQ (*P* < .001). This figure gives a more accurate picture of the course of development of VIQ and PIQ over time by displaying both longitudinal and cross-sectional data.

### Multivariable Linear Regression

Because of the significant differences found between the VIQ and PIQ and the variation of these indexes over time, we selected MLE, mode of inheritance, and ADHD as dependent variables in the regression analysis within the longitudinal groups. Because there is insufficient support in previous research for an effect of sex on intelligence scores and because we found no evidence of sex differences in intelligence scores in the cross-sectional group, we chose to exclude sex as a predictor. Considering the size of the longitudinal groups, four variables were included. MLE, mode of inheritance, and ADHD were entered into a multivariable linear regression model to examine the predictive quality of each predictor (enter method), with VIQ and PIQ scores at L2, L3, or L4 as the dependent variable, controlling for VIQ and PIQ scores at ages L1, L2, or L3, respectively.

No variable at L1 contributed significantly to VIQ or PIQ at L2. The same variables used at L2 and L3 did not predict VIQ or PIQ at L3 or L4. For L1, L2, and L3, previous performance on the FSIQ, VIQ, PIQ, and PSI predicted these outcome measures at L2, L3, and L4 respectively (see Supplementary Material, Table S6).

## Discussion

This study investigated the course of intellectual development in children with NF1 and the influence of MLE, mode of inheritance, and comorbid ADHD. To summarize,

1. We found a significant increase in of Performance IQ in the longitudinal group of children with NF1 between 11 and 16 years. PIQ appeared to be the most variable index over time. From the age of 11 years, children achieved the highest scores on the processing speed index, compared to the other indexes.

2. We found no differences in intelligence in subgroups within MLE, mode of inheritance, or comorbid ADHD.

3. Both longitudinal and cross-sectional data indicate that children between the ages of 6 and 11 years appear to face an elevated risk, given the significant differences in intelligence in favor of VIQ over PIQ. Differing cognitive levels carry the risk that a child needs to be addressed at 2 levels. Verbal IQ is generally better developed than Performance IQ in our research population, and expectations of children are often set based on language level, creating the risk of overestimation. This can especially lead to problems in upper elementary school, when decisions must be made about secondary education.

Our study contains a large data set describing longitudinal and cross-sectional intelligence characteristics in children with NF1 aged 2 years 6 months–16 years. Within the neurodevelopmental literature, research to date has focused mainly on cross-sectional groups of children and less on longitudinal follow-up over time spanning childhood, using an age-appropriate Wechsler scale. In addition, not much prospective research has been done on children with NF1 using predetermined age groups throughout childhood. Additionally, relatively little empirical research has been done on environmental influences that may contribute to phenotypic variability in longitudinal groups.

### Course of Intelligence

A prominent finding in the longitudinal analysis of children with NF1 was the significant increase in Performance IQ that we found between the ages of 11 and 15 in the longitudinal part of the study. Generally, at age 11-12 years, intelligence shows a strong association with intelligence later in adolescence, and correlations of 0.80 are found.^33^ This seems to be different for PIQ in children with NF1 aged 11 years. In the cross-sectional group, we found significant differences between VIQ and PIQ at 11 years. Performance IQ seems to improve between ages 11 and 15 years. This is in line with a previous longitudinal study.^18^ In our study, PIQ scores of the 15-year-olds in the cross-sectional group are significantly higher and the previously found difference between VIQ and PIQ disappears. In our study, there was evidence of “growing out of deficit” in PIQ. This was the case in both the cross-sectional and longitudinal groups, which reinforces the impression that there appears to be an improvement of visual-spatial reasoning over time.

### Covariables in the Relationship Between IQ and Age

The current study found no variables accounting for a significant increase in intellectual development from 11 to 15 years of age. MLE, mode of inheritance, or a comorbid diagnosis of ADHD did not significantly contribute to FSIQ, VIQ, PIQ, or PSI at L2, L3, or L4. Previous findings of a positive effect of higher socioeconomic status on intelligence^15^ were not confirmed. Compared with the study by Hou et al,^15^ our study found a smaller effect of familial NF1 and parental educational level. However, comparison of the 2 studies is complicated by differences in severity (the Hou study only includes children with plexiform neurofibromas), family circumstances (the Hou study includes relatively more children with familial NF1), age (age at first measurement in the Hou study was 12 years and in our study 3 years), and methodology (Hou: flexible ages, our study: fixed ages).” This study (2020) on 88 children with NF1 found that children who had a parent with NF1 had decreased PIQ scores compared with children with a de novo mutation. We could not confirm this in our longitudinal group of children with a familial variant (n = 36) vs children with a de novo variant (n = 84), or in our cross-sectional group of children (familial variant n = 79, de novo variant n = 169).

The percentage of children with an ADHD classification in both the cross-sectional and the longitudinal study groups is consistent with previous research,^7,22^ and ranges in our study between 34% and 57%, excluding the youngest group of children. We did find a significant difference in processing speed at age 11 years in the cross-sectional group where children with a comorbid diagnosis of ADHD score about 7 points lower than children without ADHD (*P* = .34, Cohen *d* = .420). However, these conclusions should be drawn with caution given the many comparisons made in this study. Heimgärtner et al's study^25^ showed a significant difference in FSIQ between children with NF1 and ADHD (n = 53) and NF1 only (n = 28) at the expense of the former group. We could neither confirm this in our longitudinal group of children with NF1 and ADHD (n = 46) and NF1 only (n = 69), nor in our cross-sectional group of children (NF1 and ADHD: n = 107, NF1 only: n = 179). A possible source of bias could be the use of attention-regulating medication with 38% and 80% of children in L2-3 and L3-4, respectively, and 22% of children with ADHD in the cross-sectional group as a whole using medication. As this study was noninterventional, neuropsychological testing was performed with potential current ADHD treatment. Initially, we did not target medication use. However, in an exploratory analysis of this limited group (n = 44 with prescribed ADHD medication, n = 44 no medication), we found no significant differences between children with NF1 and ADHD with or without medication on the FSIQ, VIQ, PIQ, or PSI in either the longitudinal or cross-sectional groups. Outcome scores for FSIQ, VIQ, PIQ, or PSI at respectively L1, L2, and L3 were associated with scores at L2, L3, and L4 indicating that intelligence at a younger age predicts later intelligence throughout childhood and adolescence. This expands the observation of Lorenzo and colleagues in a toddler longitudinal study.^12^ In our study, we found this not only in toddlers but also throughout childhood and adolescence.

### Strengths and Limitations

In 10 years, we have enrolled almost 400 children with NF1. We were able to include a group of 115 children with NF1 whom we followed longitudinally to better understand how intelligence develops over time. We also distinguished 4 cross-sectional groups, with relatively large sample sizes. In addition, we did not select for specific disease characteristics but studied all children with NF1 coming to both of our clinics covering a large part of the full Dutch population. Our findings are consistent with those of Ottenhoff and colleagues,^9^ whose sample partly overlaps with the current sample, as the mean intelligence for 87% was between 89 and 98 in both the cross-sectional and the longitudinal groups.

Some limitations must be addressed. First, although all children were actively referred to an outpatient clinic that has expertise in the cognition of children with NF1, referral bias cannot be ruled out. However, most pediatricians reported referring not only children with cognitive problems but also those without. Nevertheless, parents decide whether or not to follow up this referral. Compared to the Dutch population (aged 35-45 years), our sample has a relatively large proportion of people with middle-level education, which may introduce some bias. Group sizes at both ends of the distribution (3 and 15 years) are relatively smaller. For many children in the youngest age group, the diagnosis of NF1 is not yet known, which may affect referral. This has been changing in recent years because of greater awareness of clinical criteria and an increase in genetic testing. Fewer children are represented at age 15 years than at age 11 years. These 15-year-old children are now participating in secondary education, which may reduce the urgency to choose an appropriate level of education. At 15 years, there may also be less emphasis on academic learning and more on social-emotional development. Second, the sample size in the longitudinal study remains limited. Despite the careful design of the follow-up program, it has proven difficult to motivate families to visit our outpatient clinics multiple times over a longer period for extensive examinations. In order to ultimately obtain more stable results, it will be necessary to expand these samples. A third limitation is that we did not document all interventions during routine care, which may have influenced our longitudinal data. Children may have been treated over time, which may also be a source of bias. Based on our neuropsychological assessments, parents and teachers may have been advised to adjust their approach of the child. Remediation may have been recommended and implemented, or comorbid ADHD may be treated. A fourth limitation might be that the cognitive development of children with NF1 was not compared with that of a healthy control group. In this study, we relied on comparisons with rigorous Dutch norms from a large population sample. A fifth limitation could be that we used the WPPSI-III-NL and WISC-III-NL, which are currently outdated versions of the Wechsler scales in terms of norms and underlying intelligence theory, as WPPSI-IV and WISC-V do not use the PIQ -VIQ distinction anymore. This is due to the time span of the current study: 2010-2022, during this period these were the most common intelligence tests for this age. As Verbal and Performance IQ have also been used as variables in previous research, we conformed to this and did not choose for the Verbal Comprehension Index (VCI) and Visual Spatial Index (VSI). As the WPPSI-IV-NL and the WISC-V-NL are now common tests, the results of future research may be different. Finally, because this study contained many statistical comparisons, there is an inherent risk of type I errors, although we applied Bonferroni corrections. Therefore, we focused on the overall picture.

### Implications for Future Research

Future research may clarify which factors can explain the “growing out of deficit” in Performance IQ at age 15. This may be related to specific subtests within the PIQ that showed growth. Currently, the WPPSI-IV and WISC-V are used, inspired by the Cattell-Horn Carroll model. The concepts of Verbal and Performance IQ have now been replaced. Verbal IQ is replaced by the Verbal Comprehension Index (VCI). Performance IQ is replaced by the Visual Spatial Index (VSI) and the Fluid Reasoning Index (FRI). The index Processing Speed is retained and the index Working Memory (WMI) is introduced. We believe that these new tests will be even more sensitive and useful for assessing neurocognitive function in children with NF1 in the future. The neurocognitive domains of attention and executive function need to be explored, because those may be associated with an increase in Performance IQ. Finally, research into the development of intelligence and associations with later participation in work and daily life in adulthood may be highly informative.

### Implications for Clinical Practice

The Wechsler scales seem to be suitable for detecting differences in intelligence in children with NF1 and are useful worldwide. Because intelligence seems to be stable over time and we see some variation, especially around age 11 years, we might question whether we should stick to this many neuropsychological assessment ages in clinical practice. IQ measured at the age of 3 years is generally less predictive than at a later age. The question is therefore: Is this the right age to measure IQ in children with NF1? This might cause unnecessary concern for parents. However, by assessing them at this young age, we can support parents of some children in choosing between regular or special education, especially when these children are at risk of problems in development, learning, and attention. Since developmental problems can also have an impact on social-emotional well-being,^34^ assessment at the age of 3 or 4 years, just before the start of primary school, may facilitate early intervention. Between ages 6 and 11 years, there appear to be discrepancies between verbal and performance IQ. It is during this vulnerable period that a lot is expected of children at school. Therefore, we believe it is still necessary to monitor children neurocognitively during this period as they are at risk for learning and attention problems. At age 15 years, instead of a neuropsychological assessment, an initial (multidisciplinary) screening may be considered to determine if an assessment is indicated. Our results seem to indicate that young people with NF-1 need more time to reach full development. In clinical practice, it may therefore be decided to monitor these children until the age of 18-21 years. Despite the fact that the large group of 15-year-olds report relatively few intelligence problems, it is necessary to individually monitor how lower-achieving young people progress toward adulthood between the ages of 18 and 21 years. Consideration may also be given to monitoring adolescents up to the age of 18-21 years, not just in terms of intelligence but also in other neurocognitive domains and in social, emotional, and participation domains, to see how they develop over time.

Finally, previous studies have shown a negative association between cognitive variables and lower parental educational attainment, multiple cases of NF1 in the family, and ADHD. These aspects should be taken into account when determining a child's vulnerability, even though they appear to be less of a concern in the current study.

## Conclusion

With this study using longitudinal data, we gained more accurate information on development than would have been the case with cross-sectional research. This longitudinal perspective showed that there is a significant increase in PIQ in children between 11 and 15 years. PIQ appeared to be the most variable index over time. No effects of the mother's level of education (a proxy for socioeconomic status), mode of inheritance, or comorbid ADHD were found. Both longitudinal and cross-sectional data indicate that children between the ages of 6 and 11 years are at increased risk, given the significant difference in intelligence, with VIQ scoring higher than PIQ at these ages. We therefore assume that children in this age group are also at greater risk of having a disharmonious intelligence profile. Decisions about secondary education, which must be made during the final years of primary school, may be influenced by this.

Despite this variability at certain ages, intelligence appears to be a fairly stable trait over time, also in children with NF1. It is helpful for children, parents, and teachers to know that despite difficulties with Performance IQ at one point in life, children may outgrow these difficulties. Children with NF1 may mature later and take a longer time to achieve optimal functioning in PIQ. Monitoring neurocognitive development remains both necessary and useful in children with NF1.

## Supplemental Material

sj-docx-1-jcn-10.1177_08830738261416621 - Supplemental material for Intelligence Over Time in Children with Neurofibromatosis Type 1 Based on a Structured Natural History-Study Supplemental material, sj-docx-1-jcn-10.1177_08830738261416621 for Intelligence Over Time in Children with Neurofibromatosis Type 1 Based on a Structured Natural History-Study by Sandra van Abeelen, Jos G. M. Hendriksen, Anton de Louw, Marie Claire Y. de Wit, Pieter F. A. de Nijs, Rianne Oostenbrink and André B. Rietman in Journal of Child Neurology
