# Supplementary material for: Intelligence Over Time in Children with Neurofibromatosis Type 1 Based on a Structured Natural History-Study
Source: J Child Neurol. 2026 Feb 17;41(9):1311–24. doi: 10.1177/08830738261416621 (PMC13522411; doi:10.1177/08830738261416621)
Supplement: Supplementary file 1 [file sj-docx-1-jcn-10.1177_08830738261416621.docx]

**SUPPLEMENTARY MATERIALS**

**Tables S1.	Comparisons of proportions and scores between longitudinal and corresponding cross-sectional groups**

***Table S1a.	Demographic characteristics in T1 and T2.***

|  | Final group **T1**  (*N* = 83) | Longitudinal group **L1**  (*N* = 43) | Cross-sectional group **C1**  (*N* = 40) | Sig. C1 and L1 (*p*) | Final group **T2**  (*N* = 150) | Longitudinal group **L2a**  (*N* = 43) | Cross-sectional group **C2**  (*N* = 106) | Sig. C2 and L2a (*p*) |
| --- | --- | --- | --- | --- | --- | --- | --- | --- |
| Age (*SD*) |  | 3.3 (0.61) | 3.2 (0.55) |  |  | 6.4 (0.66) | 5.9 (0.73) |  |
| Sex Male  Female | 48  35 | 24  19 | 24  16 | Chi = .700 | 83  66 | 24  19 | 59  47 | Chi = .986 |
| MLE Low/Middle  High | 40  37 | 23  18 | 17  19 | Chi= .520 | 79  55 | 23  18 | 56  37 | Chi = .454 |
| CMD Yes  No | 3  80 | 0  43 | 3  37 | Fisher’s = .108 | 2  147 | 0  43 | 2  104 | Fisher’s = 1 |
| Mode of Familial  inheritance De novo | 15  66 | 9  32 | 6  34 | Chi= .604 | 38  99 | 9  32 | 29  67 | Chi= .490 |
| ADHD Yes  No | 18  65 | 12  31 | 6  34 | Chi = .154 | 6  93 | 12  31 | 44  62 | Chi = .120 |

Independent samples T-test

|  | **L1** (*N* = 43) | **C1** (*N* = 40) | Sig. C1 and L1 (*p*) | Effect size ^c^ | **L2a** (*N* = 43) | **C2** (*N* = 106) | Sig. C2 and L2a (*p*) | Effect size ^c^ |
| --- | --- | --- | --- | --- | --- | --- | --- | --- |
| FSIQ  Missing ^a^ | 90.22  2 | 89.23 | .776 | .063 | 93.79 | 89.35  1 | .064 | .338 |
| VIQ | 93.16 | 91.65 | .654 | .099 | 97.07 | 92.78 | .101 | .298 |
| PIQ | 89.09 | 89.60 | .876 | -.034 | 93.44 | 89.96 | .142 | .267 |
| PSI  Missing ^a^ | - ^b^ | - ^b^ | - ^b^ |  | 91.72 | 90.87 (*N*=102)  4 | .790 | .049 |

*Note.* Abbreviations: Age = age at the time of assessment; FSIQ = full-scale IQ; VIQ = Verbal IQ; PIQ = Performance IQ; PSI = Processing Speed Index; *N* = Number of participants; MLE = mother’s level of education; CMD = chromosomal microdeletion genotype; ADHD = attention deficit hyperactivity disorder; C1 = cross-sectional T1; L1 = longitudinal T1; C2 = cross-sectional T2; L2a = longitudinal T2. Significant p-values (**p* <.05. ***p* < .01. ****p* <.001) are in bold

^a^ Part of test not administered for unknown reason; ^b^ PSI not available for this age; ^c^ Effect size is represented by Cohen’s *d*

***Table S1b.	Demographic characteristics in T2 and T3.***

|  | Final group **T2**  (*N* = 147) | Longitudinal group **L2b**  (*N* = 41) | Cross-sectional group **C2**  (*N* = 106) | Sig. C2 and L2b (*p*) | Final group **T3**  (*N* = 148) | Longitudinal group **L3a**  (*N* = 41) | Cross-sectional group **C3**  (*N* = 108) | Sig. C3 and L3a (*p*) |
| --- | --- | --- | --- | --- | --- | --- | --- | --- |
| Age (*SD*) |  | 6.3 (0.69) | 5.9 (0.73) |  |  | 10.8 (0.79) | 11.3 (0.89) |  |
| Sex Male  Female | 76  71 | 17  24 | 59  47 | Chi = .122 | 73  75 | 17  24 | 57  51 | Chi = .217 |
| MLE Low/Middle  High | 76  51 | 20  14 | 56  37 | Chi= .887 | 83  43 | 20  14 | 63  30 | Chi = .350 |
| CMD Yes  No | 2  145 | 0  41 | 2  104 | Fisher’s = 1 | 2  146 | 0  41 | 2  106 | Fisher’s = 1 |
| Mode of Familial  inheritance De novo | 38  89 | 9  22 | 29  67 | Chi= .901 | 45  72 | 9  22 | 36  51 | Chi= .224 |
| ADHD Yes  No | 68  79 | 24  17 | 44  62 | Chi = .063 | 69  79 | 24  17 | 46  62 | Chi = .082 |

Independent samples T-test

|  | **L2b** (*N* = 41) | **C2** (*N* = 106) | Sig. C2 and L2b (*p*) | Effect size | **L3a** (*N* = 41) | **C3** (*N* = 108) | Sig. C3 and L3a (*p*) | Effect size ^c^ |
| --- | --- | --- | --- | --- | --- | --- | --- | --- |
| FSIQ  Missing ^a^ | 96.2 | 89.35 (N=105)  1 | **.005*** | .530 ^c^ | 93.85 | 90.31 | .236 | .220 |
| VIQ | 98.88 | 92.78 | **.020*** | .432 ^c^ | 96.30 | 93.59 | .344 | .176 |
| PIQ | 93.66 | 89.96 | .131 ^b^ | .280 ^d^ | 92.18 | 87.55 | .140 | .274 |
| PSI  Missing | 98.66 | 90.87 (*N*=102)  4 | **.016*** | .451 ^c^ | 101.10 | 95.38 (*N*=107)  1 | .057 | .355 |

*Note.* Abbreviations: Age = age at the time of assessment; FSIQ = Full-scale IQ; VIQ = Verbal IQ; PIQ = Performance IQ; PSI = Processing Speed Index; *N* = Number of participants; CMD = chromosomal microdeletion genotype; Mother’s level of education; ADHD = attention deficit hyperactivity disorder; C2 = cross-sectional T2; L2b = longitudinal T2; C3 = cross-sectional T3; L3a = longitudinal T3. Significant p-values (**p* <.05. ***p* < .01. ****p* <.001) are in bold

^a^ Part of test not administered for unknown reason; ^b^ Mann-Whitney U-test; ^c^ Effect size is represented by Cohen’s *d* or ^d^ by *r*

***Table S1c.	Demographic characteristics in T3 and T4.***

|  | Final group **T3**  (*N* = 139) | Longitudinal group **L3b**  (*N* = 31) | Cross-sectional group **C3**  (*N* = 108) | Sig. C3 and L3b (*p*) | Final group **T4**  (*N* = 63) | Longitudinal group **L4**  (*N* = 31) | Cross-sectional group **C4**  (*N* = 32) | Sig. C4 and L4 (*p*) |
| --- | --- | --- | --- | --- | --- | --- | --- | --- |
| Age (*SD*) |  | 11.3 (0.97) | 11.3 (0.89) |  |  | 14.97 (0.61) | 14.97 (0.74) |  |
| Sex Male  Female | 75  65 | 17  14 | 57  51 | Chi = .839 | 38  26 | 17  14 | 20  12 | Chi = .537 |
| MLE Low/Middle  High | 75  40 | 12  19 | 63  30 | Chi= .355 | 28  16 | 12  19 | 16  6 | Chi = .284 |
| CMD Yes  No | 2  138 | 0  31 | 2  106 | Fisher’s = 1 | 1  63 | 0  31 | 1  31 | Fisher’s = 1 |
| Mode of Familial  inheritance De novo | 47  64 | 10  13 | 36  51 | Chi= .856 | 19  30 | 10  13 | 8  17 | Chi= .412 |
| ADHD Yes  No | 56  84 | 10  21 | 46  62 | Chi = .301 | 21  43 | 10  21 | 11  21 | Chi = .859 |

Independent samples T-test

|  | **L3b** (*N* = 31) | **C3** (*N* = 108) | Sig. C3 and L3b (*p*) | Effect size ^b^ | **L4** (*N* = 31) | **C4** (*N* = 32) | Sig. C4 and L4 (*p*) | Effect size ^b^ |
| --- | --- | --- | --- | --- | --- | --- | --- | --- |
| FSIQ | 88.32 | 90.31 | .525 | -.130 | 89.39 | 90.78 | .744 | -.083 |
| VIQ | 91.77 | 93.59 | .558 | -.120 | 90.58 | 91.59 | .816 | -.059 |
| PIQ | 86.48 | 87.55 | .753 | -.064 | 90.35 | 92.28 | .653 | -.114 |
| PSI  Missing ^a^ | 96.81 | 95.38 (*N*=107)  1 | .654 | .092 | 95.77 | 95.78 | .999 | -.000 |

*Note.* Abbreviations: Age = age at the time of assessment; FSIQ = Full-scale IQ; VIQ = Verbal IQ; PIQ = Performance IQ; PSI = Processing Speed Index*; N* = Number of participants; CMD = chromosomal microdeletion genotype; ADHD = attention deficit hyperactivity disorder; C3 = cross-sectional T3; L3b = longitudinal T3; C4 = cross-sectional T4; L4 = longitudinal T4. Significant p-values (**p* <.05. ***p* < .01. ****p* <.001) are in bold

^a^ Part of test not administered for unknown reason; ^b^ Effect size is represented by Cohen’s *d*

**Table** **S2.	Comparison of intelligence scores between background variable subgroups in four cross-sectional groups**

| **Cohort** | **C1 (*N* = 40) ^a^** | | | | | | | | **C2 (*N* = 106)** | | | | | | | |
| --- | --- | --- | --- | --- | --- | --- | --- | --- | --- | --- | --- | --- | --- | --- | --- | --- |
| **Characteristics** | FSIQ | *p* | VIQ | *p* | PIQ | *p* | PSI | *p* | FSIQ | *p* | VIQ | *p* | PIQ | *p* | PSI | *p* |
| **Sex**  Male  Female | 90.17 (17.8)  87.81 (16.0) | .668 | 91.04 (16.2)  92.56 (17.4) | .668 | 92.50 (17.5)  85.25 (12.62) | .269 | -  - | -  - | 88.72 (12.5)  90.13 (12.4) | .626 **^a^** | 92.47 (14.6)  93.17 (14.5) | .807 | 89.78 (12.5)  90.19 (12.6) | .757 **^a^** | 87.13 (17.5)  95.57 (17.1) | **.019*** |
| **MLE**  Low-medium  High | 87.59 (15.4)  92.26 (19.2) | .657 | 91.24 (14.4)  93.68 (19.1) | .912 | 88.29 (16.6)  91.42 (17.1) | .668 | -  - | -  - | 89.20 (12.6)  91.03 (11.4) | .385 **^a^** | 92.9 (14.0)  95.59 (13.9) | .313 | 89.70 (12.7)  91.51 (12.0) | .492 | 88.91 (16.3)  93.94 (18.1) | .267 |
| **Mode of inheritance**  Familial  De novo | 85.83 (22.7)  89.82 (16.1) | .426 | 87.33 (23.9)  92.41 (15.2) | .353 | 89.17 (15.7)  89.68 (16.3) | .879 | -  - | -  - | 89.61 (12.2)  88.52 (12.8) | .703 | 91.41 (14.5)  92.67 (14.8) | .701 | 89.24 (12.1)  89.34 (12.0) | .970 | 93.61 (15.4)  90.52 (18.9) | .449 |
| **ADHD**  No  Yes | 90.50 (17.7)  82.00 (10.1) | .225 | 92.38 (17.2)  87.50 (12.47) | .531 | 91.06 (16.5)  81.33 (9.8) | .115 | -  - | -  - | 89.95 (11.7)  88.52 (13.5) | .564 | 92.52 (13.3)  92.16 (16.2) | .823 | 91.26 (12.3)  88.14 (12.6) | .205 | 90.90 (16.1)  90.84 (19.7) | .988 |
| **Cohort** | **C3 (*N* = 108)** | | | | | | | | **C4 (*N* = 32) ^a^** | | | | | | | |
| **Characteristics** | FSIQ | *p* | VIQ | *p* | PIQ | *p* | PSI | *p* | FSIQ | *p* | VIQ | *p* | PIQ | *p* | PSI | *p* |
| **Sex**  Male  Female | 91.77 (16.8)  88.71 (14.7) | .316 | 95.28 (16.2)  91.71 (13.8) | .221 | 87.60 (18.0)  87.47 (15.9) | .970 | 93.61 (16.7)  97.33 (16.0) | .242 | 86.65 (14.4)  97.67 (19.0) | .098 | 87.10 (15.8)  99.08 (18.7) | .090 | 89.85 (14.3)  96.33 (19.8) | .391 | 91.40 (16.2)  103.08 (15.9) | .109 |
| **MLE**  Low-medium  High | 91.16 (15.8)  91.30 (15.0) | .967 | 94.59 (15.0)  94.40 (12.6) | .953 | 87.32 (17.3)  89.63 (16.9) | .742 | 94.70 (15.1)  97.48 (18.0) | .442 | 93.69 (16.9)  86.50 (12.2) | .338 | 92.94 (18.7)  89.17 (16.9 | .606 | 95.94 (17.0)  86.93 (8.0) | .171 | 97.00 (18.9)  90.50 (16.9) | .236 |
| **Mode of inheritance**  Familial  De novo | 85.19 (16.2)  92.18 (14.7) | **.040*** | 89.81 (17.0)  94.20 (13.2) | .180 | 83.06 (15.6)  89.27 (17.8) | .094 | 92.22 (16.7)  96.06 (16.7) | .296 | 87.50 (18.7)  93.71 (17.7) | .466 | 87.75 (10.2)  93.71 (16.6) | .466 | 89.75 (18.9)  95.82 (17.3) | .365 | 90.88 (16.6)  102.59 (14.8) | .207 |
| **ADHD**  No  Yes | 91.76 (16.0)  88.39 (15.5) | .276 | 93.74 (15.9)  93.39 (14.2) | .906 | 89.18 (17.0)  85.33 (17.0) | .245 | 98.23 (15.4)  91.47 (17.1) | **.034*** | 88.71 (17.0)  94.73 (16.7) | .451 | 91.14 (18.5)  92.45 (16.7) | .736 | 89.14 (15.9)  98.27 (16.7) | .257 | 95.33 (14.1)  96.64 (21.9) | .735 |

*Note.* Abbreviations: FSIQ = Full-scale IQ; VIQ = Verbal IQ; PIQ = Performance IQ; PSI = Processing Speed Index*; N* = Number of participants; ADHD = attention deficit hyperactivity disorder; C1= cross-sectional T1; C2= cross-sectional T2; C3 = cross-sectional T3; C4= cross-sectional T4. Data are presented as Mean (*SD*). Significant p-values (**p* <.05. ***p* < .01. ****p* <.001) are in bold

^a^ Mann Whitney U Test was used in small sample sizes (C1, C4) or non-normality distributed variables (in C2)

**Table S3.	Independent samples t-test for comparisons between cross-sectional groups**

| **Variables** | **C1 (*N* = 40)** | **C2 (*N* = 106)** | **Sig. C1 and C2 (*p*)** | **Effect size ^d^** |
| --- | --- | --- | --- | --- |
| FSIQ  Missing ^a^ | 89.23 | 89.35  1 | .966 | -.009 |
| VIQ | 91.65 | 92.78 | .686 | -.075 |
| PIQ | 89.60 | 89.96 | .888 | -.012 |
| PSI  Missing ^a^ | 94.67 ^b^  (*N*=9) | 90.87  (*N*=102)  4 | - ^b^ | - ^b^ |
|  | **C2 (*N* = 106)** | **C3 (*N* = 108)** | **Sig. C2 and C3 (*p*)** | **Effect size** |
| FSIQ  Missing ^a^ | 89.35  1 | 90.31 | .618 | -.068 ^d^ |
| VIQ | 92.78 | 93.59 | .690 | -.055 ^d^ |
| PIQ | 89.96 | 87.55 | .233 ^c^ | -.082 ^e^ |
| PSI  Missing ^a^ | 90.87  4 | 95.38  1 | .057 | -.265 ^d^ |
|  | **C3 (*N* = 108)** | **C4 (*N* = 32)** | **Sig. C3 and C4 (*p*)** | **Effect size ^d^** |
| FSIQ | 90.31 | 90.78 | .885 | -.029 |
| VIQ | 93.59 | 91.59 | .528 | .127 |
| PIQ | 87.55 | 92.28 | .165 | -.281 |
| PSI  Missing ^a^ | 95.38  1 | 95.78 | .905 | -.024 |

*Note:* Abbreviations: FSIQ = Full-scale IQ; VIQ = Verbal IQ; PIQ = Performance IQ; PSI = Processing Speed Index*; N* = Number of participants; C1= cross-sectional T1; C2= cross-sectional T2; C3 = cross-sectional T3; C4= cross-sectional T4.

Significant p-values (**p* <.05. ***p* < .01. ****p* <.001) are in bold

^a^ Part of test not administered for unknown reason

^b^ PSI not available for this age

^c^ Mann-Whitney U test

^d^ Effect size is represented by Cohen’s *d* or ^e^ by *r*

**Table S4.	Intelligence within longitudinal groups**

| **Cohort (*N*, M age in years)** | **FSIQ** | **VIQ** | **PIQ** | **PSI** | ***p* ^d^** | **Effect size ^e^** |
| --- | --- | --- | --- | --- | --- | --- |
| **L1 ^a^** (43, 3;3) | 90.22 (14.35) ^b^ | 93.16 (14.14) | 89.09 (13.48) | - ^c^ |  |  |
| **L2a ^a^** (43, 5;9) | 94.20 (14.97) ^b^ | 97.07 (14.04) | 93.44 (14.37) | 91.72 (16.94) | VIQ-PSI: .**027*** | .350 |
| **L2b ^a^** (41, 6;4) | 96.20 (15.00) | 98.88 (13.06) | 93.66 (15.02) | 98.66 (16.19) | VIQ-PIQ: **<.009**** | .426 |
| **L3a ^a^** (41, 10;8) | 93.80 (16.44) | 96.22 (16.03) | 92.17 (16.42) | 100.93 (15.15) | PIQ-PSI: **<.001***** | -.659 |
| **L3b ^a^** (31, 11;3) | 88.32 (13.64) | 91.77 (15.13) | 86.48 (14.16) | 96.81 (12.05) | PIQ-PSI: **<.001***** | -.935 |
| **L4 ^a^** (31, 14;9) | 89.39 (16.78) | 90.58 (16.82) | 90.35 (17.30) | 95.77 (13.73) | PIQ-PSI: **<.026*** | -.421 |

^a^ L1 longitudinal group 3 years, L2a longitudinal group 6 years, L2b longitudinal group 6 years, L3a longitudinal group 11 years, L3b longitudinal group 11 years; L4 longitudinal group 15 years

^b^ *N* = 41, data of 2 patients missing

^c^ PSI not available at this age

^d^ Significant differences between subscales of intelligence

^e^ Effect size is represented by Cohen’s *d*

All data are presented as Mean (*SD*)

*Significant p-values are in bold; *p* <.05. ***p* < .01. ****p* <.001. After correction for multiple comparisons α was reduced to 0.017 (*p* < 0.05): 0.05/3.

**Table S5.	Paired-samples t-tests in longitudinal groups**

|  | **Longitudinal groups** | | ***p*** | **Effect size ^d^** |  | **Longitudinal groups** | | ***p*** | **Effect size ^d^** |  | **Longitudinal groups** | | ***p*** | **Effect size ^d^** |
| --- | --- | --- | --- | --- | --- | --- | --- | --- | --- | --- | --- | --- | --- | --- |
| **L1-2**  *N* = 43  3;3-5;9 y ^a^ (*SD 0.6-0.7)* | **L1** | **L2a** |  |  | **L2-3**  *N* = 41  6;4-10;8 y ^a^  (*SD 0.7-0.8)* | **L2b** | **L3a** |  |  | **L3-4**  *N* = 31  11;3-14;9 y ^a^  *(SD 1-0.7)* | **L3b** | **L4** |  |  |
|  | **FSIQ** | |  |  |  | **FSIQ** | |  |  |  | **FSIQ** | |  |  |
|  | 90.22 (14.35) ^b^ | 94.20 (14.97) ^b^ | **.023*** | - .369 |  | 96.20 (15.00) | 93.80 (16.44) | .179 | .214 |  | 88.32 (13.64) | 89.39 (16.78) | .422 | - .146 |
|  | **VIQ** | |  |  |  | **VIQ** | |  |  |  | **VIQ** | |  |  |
|  | 93.16 (14.14) | 97.07 (14.04) | **.022*** | - .361 |  | 98.88 (13.06) | 96.22 (16.03) | .121 | .247 |  | 91.77 (15.13) | 90.58 (16.82) | .420 | .147 |
|  | **PIQ** | |  |  |  | **PIQ** | |  |  |  | **PIQ** | |  |  |
|  | 89.09 (13.48) | 93.44 (14.37) | **.034*** | - .334 |  | 93.66 (15.02) | 92.17 (16.42) | .518 | .102 |  | 86.48 (14.16) | 90.35 (17.30) | **.017*** | - .454 |
|  | **PSI** | |  |  |  | **PSI** | |  |  |  | **PSI** | |  |  |
|  | - ^c^ | 91.72 (16.94) | - | - |  | 98.66 (16.19) | 100.93 (15.15) | .362 | - .144 |  | 96.81 (12.05) | 95.77 (13.73) | .663 | .079 |

*Note.* Abbreviations: FSIQ = Full-scale IQ; VIQ = Verbal IQ; PIQ = Performance IQ; PSI = Processing Speed Index*; N* = Number of participants; L1-2 consists of longitudinal groups L1 and L2a (3 and 6 years); L2-3 consists of longitudinal groups L2b and L3a (6 and 11 years); L3-4 consists of longitudinal groups L3b and L4 (11 and 15 years)

Data are presented as Mean (*SD*). Significant p-values (**p* <.05. ***p* < .01. ****p* <.001) are in bold

^a^ Mean ages in years (*SD*)

^b^ *N* = 41 because parts of test of 2 patients were not administered

^c^ PSI not available at this age

^d^ Effect size is represented by Cohen’s *d*

*Significant p-values are in bold; *p* <.05. ***p* < .01. ****p* <.001

**Table S6.	Results multivariable linear regression analysis in longitudinal groups**

| **FSIQ 6 years** | | | | |
| --- | --- | --- | --- | --- |
| **Variables** | **β Coefficient** | **Standard error** | ***t*-value** | ***p*-value** |
| FSIQ 3 years | .772 | .135 | 5.761 | **< .001***** |
| MLE | .065 | 3.587 | .509 | .614 |
| ADHD | -.076 | 3.803 | -.652 | .519 |
| Mode of inheritance | .237 | 3.995 | 1.855 | .073 |
| **Note: *N* = 43. *R*-squared = .551, *F* = 10.109, *p* = < .001***** | | | | |
| **VIQ 6 years** | | | | |
| **Variables** | **β Coefficient** | **Standard error** | ***t*-value** | ***p*-value** |
| VIQ 3 years | .652 | .125 | 4.705 | **< .001***** |
| MLE | .182 | 3.318 | 1.380 | .176 |
| ADHD | .034 | 3.572 | .271 | .788 |
| Mode of inheritance | .098 | 3.893 | .715 | .480 |
| **Note: *N* = 43, *R*-squared = .471, *F* = 7.570, *p* = < .001***** | | | | |
| **PIQ 6 years** | | | | |
| **Variables** | **β Coefficient** | **Standard error** | ***t*-value** | ***p*-value** |
| PIQ 3 years | .607 | .159 | 4.091 | **< .001***** |
| MLE | .033 | 4.264 | .223 | .825 |
| ADHD | -.160 | 4.355 | -1.187 | .243 |
| Mode of inheritance | .148 | 4.556 | 1.047 | .302 |
| **Note: *N* = 43, *R*-squared = .384, *F* = 5.2947.570, *p* = .002**** | | | | |
|  | | | | |
| **FSIQ 11 years** | | | | |
| **Variables** | **β Coefficient** | **Standard error** | ***t*-value** | ***p*-value** |
| FSIQ 6 years | .750 | .181 | 5.303 | **< .001***** |
| MLE | .229 | 4.717 | 1.605 | .124 |
| ADHD | -.140 | 4.612 | -1.000 | .329 |
| Mode of inheritance | .000 | 5.253 | -.003 | .997 |
| **Note: *N* = 41, *R*-squared = .611, *F* = 7.845, *p* = < .001***** | | | | |
| **VIQ 11 years** | | | | |
| **Variables** | **β Coefficient** | **Standard error** | ***t*-value** | ***p*-value** |
| VIQ 6 years | .758 | .167 | 5.543 | **< .001***** |
| MLE | .282 | 4.366 | 2.054 | .053 |
| ADHD | -.241 | 4.257 | -1.804 | .086 |
| Mode of inheritance | .001 | 4.870 | .009 | .993 |
| **Note: *N* = 41, *R*-squared = .645, *F* = 9.080, *p* = < .001***** | | | | |
| **PIQ 11 years** | | | | |
| **Variables** | **β Coefficient** | **Standard error** | ***t*-value** | ***p*-value** |
| PIQ 6 years | .613 | .214 | 3.462 | **.002**** |
| MLE | .083 | 6.034 | .463 | .648 |
| ADHD | .043 | 5.958 | .242 | .811 |
| Mode of inheritance | -.019 | 6.672 | -.109 | .915 |
| **Note: *N* = 41, *R*-squared = .386, *F* = 3.142, *p* = .037*** | | | | |
| **FSIQ 15 years** | | | | |
| **Variables** | **β Coefficient** | **Standard error** | ***t*-value** | ***p*-value** |
| FSIQ 11 years | 1.071 | .156 | 8.415 | **< .001***** |
| MLE | -.182 | 3.159 | -1.905 | .081 |
| Mode of inheritance | .135 | 3.755 | 1.207 | .251 |
| ADHD | .100 | 4.150 | .874 | .399 |
| **Note: *N* = 31, *R*-squared = .905, *F* = 28.447, *p* = < .001***** | | | | |
| **VIQ 15 years** | | | | |
| **Variables** | **β Coefficient** | **Standard error** | ***t*-value** | ***p*-value** |
| VIQ 11 years | .917 | .163 | 5.935 | **< .001***** |
| MLE | -.130 | 4.335 | -1.068 | .306 |
| Mode of inheritance | -.059 | 4.982 | -.432 | .673 |
| ADHD | -.010 | 5.228 | -.077 | .940 |
| **Note: *N* = 31, *R*-squared = .851, *F* = 17.114, *p* = < .001***** | | | | |
| **PIQ 15 years** | | | | |
| **Variables** | **β Coefficient** | **Standard error** | ***t*-value** | ***p*-value** |
| PIQ 11 years | .874 | .235 | 5.095 | **.002**** |
| MLE | -.225 | 4.524 | -1.473 | .648 |
| Mode of inheritance | .116 | 4.510 | .774 | .811 |
| ADHD | .022 | 5.480 | .130 | .915 |
| **Note: *N* = 31, *R*-squared = .786, *F* = 11.025, *p* = < .001***** | | | | |

*Note.* Abbreviations: FSIQ = Full-scale IQ; VIQ = Verbal IQ; PIQ = Performance IQ; MLE = mother’s level of education; ADHD = attention deficit hyperactivity disorder

Enter method was used. *N* = Number of participants*, F* = F-ratio, β is Unstandardized Coefficient

*Significant p-values are in bold; *p* <.05. ***p* < .01. ****p* <.001

**Table S7.	Results multivariable linear regression analysis in longitudinal groups**

Comparison of WPPSI-III and WISC-III Indices and Subtests

| **Feature** | **WPPSI-III (Age 2:6–7:3)** | | **WISC-III (Age 6–16)** |
| --- | --- | --- | --- |
|  | **2;6-3;11** | **4;0-7;11** |  |
| Full Scale IQ | Yes | Yes | Yes |
| Core Indices | VIQ, PIQ | VIQ, PIQ, PSI | VIQ, PIQ, PSI |
| Total Subtests | 4 core | 7 core | 10 core |
| Processing Speed Components | - | Symbol Search | Coding, Symbol Search |

Core subtests breakdown:

**WPPSI-III:**

- **2;6-3;11**
- Verbal IQ: Information, Receptive Vocabulary.
- Performance IQ: Block Design, Object Assembly.
- **4;0-7;11**
- Verbal IQ: Information, Vocabulary, Word Reasoning.
- Performance IQ: Block Design, Matrix Reasoning, Picture Concepts.
- Processing Speed Index: Symbol Search.

**WISC-III:**

- Verbal IQ: Information, Similarities, Arithmetic, Vocabulary, Comprehension (Optional: Digit Span).
- Performance IQ: Picture Completion, Coding (Digit Symbol), Picture Arrangement, Block Design, Object Assembly (Optional: Symbol Search, Mazes).
- Processing Speed Index: Coding, Symbol Search.
